# The role of personality in health care use: Results of a population-based longitudinal study in Germany

**DOI:** 10.1371/journal.pone.0181716

**Published:** 2017-07-26

**Authors:** André Hajek, Jens-Oliver Bock, Hans-Helmut König

**Affiliations:** Department of Health Economics and Health Services Research, University Medical Center Hamburg-Eppendorf, Hamburg, Germany; Hebrew University, ISRAEL

## Abstract

**Objective:**

To determine the role of personality in health care use longitudinally.

**Methods:**

Data were derived from the German Socio-Economic Panel (GSOEP), a nationally representative, longitudinal cohort study of German households starting in 1984. Concentrating on the role of personality, we used data from the years 2005, 2009 and 2013. Personality was measured by using the GSOEP Big Five Inventory (BFI-S). Number of physician visits in the last 3 months and hospital stays in the last year were used as measures of health care use.

**Results:**

Adjusting for predisposing factors, enabling resources, and need factors, fixed effects regressions revealed that physician visits increased with increasing neuroticism, whereas extraversion, openness to experience, agreeableness and conscientiousness did not affect physician visits in a significant way. The effect of self-rated health on physician visits was significantly moderated by neuroticism. Moreover, fixed effects regressions revealed that the probability of hospitalization in the past year increased with increasing extraversion, whereas the other personality factors did not affect this outcome measure significantly.

**Conclusion:**

Our findings suggest that changes in neuroticism are associated with changes in physician visits and that changes in extraversion are associated with the probability of hospitalization. Since recent studies have shown that treatments can modify personality traits, developing interventional strategies should take into account personality factors. For example, efforts to intervene in changing neuroticism might have beneficial effects for the healthcare system.

## Introduction

### Background

To understand factors that influence people’s health care use has been an important research area for several decades. For example, since the 1960s various theoretical frameworks have been developed to identify determinants of health care use [1]. Many studies examining patterns of health care use referred to the Andersen Behavioral Model [2] as a theoretical framework for their empirical analyses. Andersen and Newman distinguish between three main components of determinants of health care use: predisposing factors, enabling resources, and need factors. Predisposing factors comprise all socio-demographics, social structure and health beliefs. An enabling resource can be on the personal, family or community level, while need factors are either perceived or evaluated. There is to a certain extent a heterogeneity of variables operationalizing the three main components of Andersen’s Behavioral Model [3]. For example, age, gender, marital status, educational level, or ethnicity defined the predisposing factors in many studies. Yet, none of the studies between 1998 and 2011 reviewed by Babitsch and colleagues [3] considered personality traits as predisposing nor as independent factors potentially explaining patterns of health care use. However, Andersen [2] suggested that psychological factors could be taken into consideration in his model.

While some cross-sectional studies exist, mainly investigating the association between personality and *mental* health services use [4–6], only one recent cross-sectional study by Friedman and colleagues [7] investigated the association between personality and health care use *in a broader sense* (e.g. covering emergency department use, hospital inpatient, or rehabilitation hospital). The study by Friedman and colleagues investigated the association between personality and health care use in old age in the United States and reported important associations. They found, for example, that increased neuroticism was associated with the use of various health care services including, for example, a greater likelihood of use of emergency departments. Other personality traits were associated with specific services as well, in some cases explaining up to 30% for the variance in health care use.

However, despite this first study drawing the attention towards the relationship of personality and health care use, it remains an open question how personality is related to health care use longitudinally. Yet a longitudinal examination is necessary to receive insights into causality. Equally, there is no study based on a representative adult population, examining the impact of personality on health care use. In other words, as already pointed out by Friedman and colleagues [7], the psychological literature in this area is still underdeveloped.

### Personality

While it must be acknowledged that other models exist [8, 9], personality is most commonly divided into five big traits [10, 11], namely *Agreeableness*, *Conscientiousness*, *Extraversion*, *Neuroticism and Openness to Experience*. (i) Agreeableness refers to the tendency to get along well with others and is associated with altruism or modesty. (ii) Conscientiousness refers to the extent to which an individual is careful, reliable and persevering. (iii) Extraversion refers to the tendency to experience positive emotions and to have a positive outlook on life. In general, extraverts are talkative, sociable, outgoing, enthusiastic, and energetic. (iv) Neuroticism refers to the tendency to experience negative emotional states including anxiety, depression or anger. (v) Openness to experience refers to the tendency to be open-minded, imaginative, and curious.

As indicated by some recent studies [12, 13], personality changes at least as much as economic factors, including in older adulthood [14–16]. For example, using data from the *Household*, *Income and Labour Dynamics in Australia* (HILDA; years 2005 and 2009; 8,625 individuals), Boyce et al. [13] have demonstrated that personality varies at least as much as economic factors (e.g., income or unemployment) [13]. Moreover, it has been shown that treatments can modify personality traits [17]. Therefore, personality can be viewed as time-varying variable that is potentially modifiable.

### Aim and hypotheses

Consequently, we aimed at examining whether personality affects health care use longitudinally. We hypothesize that higher neuroticism is associated with higher health care use. This is supported by several studies showing that neurotic individuals use more mental health services [4, 5]. Individuals high in neuroticism experience more negative emotions. This in turn could lead to more health care use such as physician visits.

Moreover, we hypothesize that extraverts talk with their friends about their ailments. Thus, their social networks might encourage them to use health care services. Another explanation might be that higher extraversion is positively associated with smoking [18], alcohol use [19] and injury-prone behavior [20].

As already found (with any custodial nursing home use and emergency department visits as outcome variables) and discussed by Friedman et al. [7], we hypothesize that conscientiousness is negatively associated with health care use. This might be explained by the negative association between conscientiousness and accidents as well as the positive association with health related behavior [21, 22]. Thus, it appears plausible that conscientiousness is negatively related to health care use.

The impact of agreeableness, and openness to experience on health care use were analyzed in an explorative way. Moreover, we examined whether personality moderates the impact of need factors on health care use. This moderation analysis was conducted because it appears plausible that the association between need factors and health care use varies by personality factors such as neuroticism. For example, compared with individuals scoring low in neuroticism, individuals scoring high in neuroticism might be more reactive to poor health (e.g., more intense feelings of pain [23].

## Methods

### Sample

Data were derived from the German Socio-Economic Panel (GSOEP), located at the German Institute for Economic Research, DIW Berlin. Beginning in 1984, the GSOEP is a nationally representative, longitudinal study of individuals living in Germany. Nearly 11,000 households and more than 20,000 individuals are interviewed on an annual basis. The data provide information on all household members (consisting of Germans living in the Old and New German States, Foreigners, and recent Immigrants to Germany). For example, topics examined include health, satisfaction, employment, and earnings. Very high wave-to-wave reinterview response rates for the GSOEP have been reported [24]. Moreover, survey attrition is quite low [25]. Individuals were asked about their personality in 2005, 2009 and 2013. Consequently, we restricted our analyses to these waves. More details regarding the sampling frame and survey design were reported elsewhere [26]. The GSOEP is approved as being in accordance with the standards of the Federal Republic of Germany for lawful data protection. Informed consent was obtained from all participants. An ethical approval was not obtained because criteria for the need of an ethical statement were not met (risk for the respondents, lack of information about the aims of the study, examination of patients). However, the German Council of Science and Humanities (Wissenschaftsrat) evaluated the German Socio-Economic Panel (GSOEP) at the Deutsches Institut für Wirtschaftsforschung, (DIW), Berlin. The German Council of Science and Humanities approved the GSOEP.

### Dependent variables

Health care was assessed by using the hospital treatment in the past twelve months and the number of outpatient physician visits in the last three months (both self-rated):

- Hospital stay for at least one night (0 = no; 1 = yes)
- Number of physician visits

### Independent variables

The short version of the Big Five Inventory (BFI-S) [27] consists of 15 items based on the original 44-item Big Five Inventory [28]. Thus, the BFI-S assesses the personality by means of three items per dimension. Individuals rated the statements on a 7-point Likert scale (from 1 = “does not apply to me at all” to 7 = “applies to me perfectly”). The psychometric properties of the BFI-S are satisfactory [29].

As for predisposing factors, age, gender, marital status (Married, living together with my spouse; others (Married, living (permanently) separated from my spouse; single; divorced; widowed), unemployment (Ref.: unemployed) and educational level (International Standard Classification of Education (ISCED-97) [30]) (with three categories: low (ISCED 0–2), medium (ISCED 3–4) and high (ISCED 5–6)) were included.

As for enabling resources, income was quantified by using the (log) square root equivalence scale (dividing the total household net income by the square root of the household’s members). Thus, synergy effects were taken into consideration.

As for need factors, subjective health and morbidity was included in our main model. Subjective health was quantified by using the self-rated health (1 = “bad” to 5 = very good”). Moreover, participants were asked whether they were “legally classified as handicapped or capable of gainful employment only to a reduced extent due to medical reasons” (no; yes). Hence, disability was used as a proxy for morbidity [31–33].

In additional models, morbidity was measured by using a count score of chronic illnesses (from 2009 onwards: diabetes, asthma, cardiac disease, cancer, heart attack, migraine, high blood pressure and dementia) instead of disability. Consequently, we solely examined changes between 2009 and 2013 in additional analysis.

### Statistical analysis

In a first step, sample characteristics for individuals included in fixed effects regressions were reported. In a second step, pairwise correlations were reported. Bonferroni-adjusted significance levels were used (‘bonferroni’ option in the ‘pwcorr’-command). The ‘bonferroni’ option makes the Bonferroni adjustment to calculated significance levels. Third, regression analysis was performed.

We used fixed effects (FE) regressions to estimate the impact of time-varying predictors on health care use. If time-constant unobserved factors (such as genetic disposition) are correlated with predictors in a systematic manner, random effects (RE) regression techniques lead to estimates that are inconsistent [34, 35]. In contrast, FE regressions provide estimates that are consistent—even under the assumption of time-constant factors that are systematically associated with the regressors. Therefore, FE regressions were used (as indicated by Sargan Hansen tests).

FE regressions merely uses changes within individuals over time. That is why the FE-estimator is sometimes also called the ‘within-estimator’. Thus, the FE-estimator solely uses time-varying variables as independent variables. In other words: Time-constant variables including gender, race or country of origin cannot be included as independent variables in FE regressions.

The regressors of physician visits (count data) were estimated by using FE poisson regressions, whereas the regressors of hospital stays (binary) were estimated by using conditional FE logistic regressions. The level of significance was set at α = .05. Statistical analysis was performed using Stata 14.0 (Stata Corp., College Station, Texas).

## Results

### Sample characteristics

The pooled (2005, 2009 and 2013) median for physician visits in the last 3 months was 2, 71.6% of participants had at least one physician visit in the last 3 months. About 12.6% were hospitalized during the 12 months preceding the interview.

As our interest lies in changes within individuals over time, individuals were only included in the sample if they had changes in the outcome variable between 2005, 2009 and 2013. Descriptive statistics for individuals included in FE regressions with physician visits and hospital stay as outcome variable are displayed in Table 1. As for the time-constant variable sex (not included in FE regressions), the majority was female (54.3%).

**Table 1:** Sample characteristics for individuals included in fixed effects regressions (with physician visits and hospital stay as outcome variable, with 2005, 2009 and 2013, pooled).

		Physician visits	Hospital stay
Time-constant variables (not included as independent variable in FE regressions)	Female: N (%)	20,206 (54.3)	5,740 (55.1)
Predisposing factors	Age (in years): Mean (SD)	51.6 (16.7)	55.2 (16.8)
	Married, living together with spouse: N (%)	13,594 (36.6)	3,534 (33.9)
	Unemployed: N (%)	2,079 (5.6)	627 (6.0)
	Low education	5,425 (14.6)	1,608 (15.4)
	Medium education	19,987 (53.7)	5,628 (54.0)
	High education	11,773 (31.7)	3,188 (30.6)
Enabling resources	Equivalence income	2,138.5 (1,515.2)	2,020.7 (1,482.7)
Need factors	Self-rated health (from “very good” to “very bad”)	2.7 (0.9)	3.0 (1.0)
	Not severely disabled: N (%)	31,950 (85.9)	8,017 (76.9)
Personality	Neuroticism (higher values indicate higher neuroticism)	11.7 (3.6)	12.0 (3.6)
	Extraversion (higher values indicate higher extraversion)	14.4 (3.4)	14.3 (3.4)
	Openness to experience (higher values indicate higher openness)	13.4 (3.6)	13.3 (3.6)
	Agreeableness (higher values indicate higher agreeableness)	16.2 (2.9)	16.2 (3.0)
	Conscientiousness (higher values indicate higher conscientiousness)	17.6 (2.7)	17.7 (2.7)
	Observations	37,185	10,424

As for the individuals included in FE regression analysis with physician visits as outcome measure, mean age was 51.6 years (±16.7 years), ranging from 17 to 103 years. The majority had a medium education (53.7%) and had a mean net equivalence income of €2,135.5 (±€1,512.2). Furthermore, only 5.6% were unemployed. The mean self-rated health was 2.7 (±0.9) and most of the individuals were not severely disabled (85.9%).

As for personality factors, the mean neuroticism score was 11.7 (±3.6), the mean extraversion score was 14.4 (±3.4), the mean openness to experience score was 13.4 (±3.6), the mean agreeableness score 16.2 (±2.9), and the mean conscientiousness score was 17.6 (±2.7). All personality factors ranged between 3 and 21. In total, the descriptive statistics for individuals included in FE regressions with hospital stay as outcome measure were similar. However, the mean age was 55.2 years (±16.8 years) and the mean self-rated health was 3.0 (±1.0). Furthermore, 76.9% were not severely disabled.

### Correlations

To get a better understanding of our data, pairwise correlations were reported in Table 2. While physician visits were positively associated with neuroticism, physician visits were negatively associated with extraversion and conscientiousness. Physician visits were not significantly associated with openness to experience and agreeableness.

**Table 2:** Pairwise correlations (with Bonferroni-adjusted significance level).

	(1) Physician visits	Hospital stay (Ref.: No)	Age (in years)	Other marital statuses (Ref.: Married, living together with spouse)	Education (ISCED-97)	Employment status (Ref.: Currently unemployed)	(Log) equivalence income	Self-rated health (from 'very good' to 'bad')	Severely disabled (Ref.: Not severely disabled)	Neuroticism (higher values indicate higher neuroticism)	Extraversion (higher values indicate higher extraversion)	Openness to experience (higher values indicate higher openness)	Agreeableness (higher values indicate higher agreeableness)	Conscientiousness (higher values indicate higher conscientiousness)
Physician visits	1													
Hospital stay (Ref.: No)	0.230***	1												
Age (in years)	0.175***	0.133***	1											
Other marital statuses (Ref.: Married, living together with spouse)	0.0383***	0.0233***	0.305***	1										
Education (ISCED-97)	-0.0161**	-0.0164**	0.114***	0.158***	1									
Employment status (Ref.: Currently unemployed)	-0.00579	-0.00671	0.107***	0.0834***	0.0835***	1								
(Log) equivalence income	-0.0436***	-0.0550***	-0.00399	0.174***	0.323***	0.247***	1							
Self-rated health (from 'very good' to 'bad')	0.384***	0.227***	0.391***	0.0963***	-0.0571***	-0.0482***	-0.132***	1						
Severely disabled (Ref.: Not severely disabled)	0.261***	0.178***	0.265***	0.0423***	-0.0443***	0.00471	-0.0624***	0.362***	1					
Neuroticism (higher values indicate higher neuroticism)	0.160***	0.0701***	0.0315***	0.0281***	-0.0920***	-0.0610***	-0.119***	0.293***	0.102***	1				
Extraversion (higher values indicate higher extraversion)	-0.0233***	-0.0110	-0.105***	-0.0437***	0.00557	0.00574	0.0493***	-0.128***	-0.0524***	-0.161***	1			
Openness to experience (higher values indicate higher openness)	-0.0117	-0.0212***	-0.0742***	-0.0370***	0.151***	0.0272***	0.122***	-0.107***	-0.0377***	-0.0636***	0.373***	1		
Agreeableness (higher values indicate higher agreeableness)	-0.00202	-0.000355	0.0689***	-0.00693	-0.0129	0.00927	-0.0547***	-0.0438***	-0.0125	-0.105***	0.0817***	0.122***	1	
Conscientiousness (higher values indicate higher conscientiousness)	-0.0162**	-0.00851	0.130***	0.126***	0.0529***	0.0192***	0.0165**	-0.0419***	-0.0120	-0.0891***	0.176***	0.151***	0.299***	1
Observations	66008													

^+^ *p* < 0.10,

* *p* < 0.05,

** *p* < 0.01,

*** *p* < 0.001

The probability of hospitalization was positively associated with neuroticism, whereas it was negatively associated with openness to experience. However, the probability of hospitalization was not significantly associated with extraversion, agreeableness and conscientiousness.

Furthermore, it is worth noting that pairwise correlations (not displayed in Table 2) between chronic conditions and personality factors were as follows: neuroticism (r = .18, p <.001), extraversion (r = -.06, p <.001), openness to experience (r = -.01, p = .86), agreeableness (r = .01, p = .39) and conscientiousness (r = .01, p = 1.0).

### Main regression analysis

FE regressions showed that physician visits increased with increasing neuroticism (β = .01, p <.001) and decreasing conscientiousness (β = -.01, p <.05), whereas the other personality factors (extraversion, openness to experience, and agreeableness) did not affect physician visits (Table 3). Furthermore, FE regressions revealed that physician visits increased with decreasing age, deteriorating self-rated health, and the onset of disability.

**Table 3:** Predictors of physician visits. Results of fixed effects poisson regression (Wave 2005, wave 2009, and wave 2013).

	Independent variables	Physician visits
Predisposing factors	Age (in years)	-0.00737***
		(0.00198)
	Other marital statuses (Ref.: Married, living together with spouse)	0.0489
		(0.0343)
	Medium education (ISCED-97, Ref.: Low education)	-0.0463
		(0.0590)
	High education (ISCED-97, Ref.: Low education)	0.0141
		(0.0852)
	Employment status (Ref.: Currently unemployed)	-0.0380
		(0.0411)
Enabling resources	(Log) equivalence income	-0.0104
		(0.0291)
Need factors	Self-rated health (from ‘very good’ to ‘bad’)	0.423***
		(0.0117)
	Severely disabled (Ref.: Not severely disabled)	0.163***
		(0.0359)
Personality	Neuroticism (higher values indicate higher neuroticism)	0.0136***
		(0.00314)
	Extraversion (higher values indicate higher extraversion)	0.00125
		(0.00357)
	Openness to experience (higher values indicate higher openness)	-0.00447
		(0.00315)
	Agreeableness (higher values indicate higher agreeableness)	0.00414
		(0.00370)
	Conscientiousness (higher values indicate higher conscientiousness)	-0.00928*
		(0.00406)
	Observations	37,185
	Number of Individuals	14,462

Comments: Poisson regression coefficients were reported; Cluster-robust standard errors in parentheses;

*** p<0.001,

** p<0.01,

* p<0.05,

^+^ p<0.10

The main model was extended by adding interaction terms between self-rated health and personality factors (neuroticism, extraversion, openness to experience, agreeableness, and conscientiousness). The effect of self-rated health on physician visits was significantly moderated by neuroticism (i.e., self-rated health x neuroticism, β = -.01, p <.05), whereas the other interaction terms (self-rated health x (extraversion, openness to experience, agreeableness, and conscientiousness)) did not achieve statistical significance.

FE regressions showed that the probability of hospitalization in the previous year increased with increasing extraversion (OR: 1.02, p <.05), whereas the other personality factors did not affect this outcome variable significantly (Table 4; personality coefficients based on z-scores: see S1 and S2 Tables). Moreover, FE regressions revealed that the probability of hospitalization increased with increasing age, increasing income, worsening self-rated health, and the onset of disability.

**Table 4:** Predictors of hospital stay. Results of conditional fixed effects logistic regression (Wave 2005, wave 2009, and wave 2013).

	Variables	Hospital
Predisposing factors	Age (in years)	1.024***
		(1.012–1.036)
	Other marital statuses (Ref.: Married, living together with spouse	1.170
		(0.969–1.413)
	Medium education (ISCED-97, Ref.: Low education)	0.796
		(0.509–1.244)
	High education (ISCED-97, Ref.: Low education)	1.320
		(0.692–2.515)
	Employment status (Ref.: Currently unemployed)	0.993
		(0.791–1.247)
Enabling resources	(Log) equivalence income	0.852*
		(0.727–0.999)
Need factors	Self-rated health (from ‘very good’ to ‘bad’)	1.670***
		(1.564–1.784)
	Severely disabled (Ref.: Not severely disabled)	1.249*
		(1.047–1.491)
Personality	Neuroticism (higher values indicate higher neuroticism)	1.014
		(0.997–1.032)
	Extraversion (higher values indicate higher extraversion)	1.024*
		(1.004–1.045)
	Openness to experience (higher values indicate higher openness)	0.982+
		(0.964–1.000)
	Agreeableness (higher values indicate higher agreeableness)	1.010
		(0.989–1.031)
	Conscientiousness (higher values indicate higher conscientiousness)	0.988
		(0.966–1.009)
	Observations	10,424
	Number of Individuals	3,947
	Pseudo R^2^	0.048

Comments: Odd Ratios were reported; 95% Confidence intervals in parentheses;

*** p<0.001,

** p<0.01,

* p<0.05,

^+^ p<0.10

### Additional model

The robustness of our variables of interest (in terms of significance and effect size) was tested by comparing the main model with an additional model. In this additional model, disability was replaced by the number of chronic conditions. It is worth emphasizing that the number of chronic conditions was included from 2009 onwards. Therefore, only changes from 2009 to 2013 (main model: 2005, 2009 and 2013) were analyzed in our additional model.

FE regressions showed that the effect of neuroticism on physician visits remained virtually the same (β = .01, p <.01), whereas the effect of conscientiousness on physician visits vanished. Furthermore, FE regressions showed that the effect of extraversion on the probability of hospitalization remained almost the same (OR: 1.03, p <.05), but there was an additional effect of neuroticism (OR: 1.03, p <.05).

## Discussion

### Main findings

We aimed at examining whether personality affects health care use longitudinally (GSOEP data). Moreover, we aimed at examining whether personality moderates the impact of need factors on health care use.

*Pairwise correlations* showed that neuroticism, extraversion and conscientiousness were correlated with physician visits and neuroticism as well as openness to experience were associated with hospital stays.

Adjusting for predisposing factors, enabling resources, and need factors, FE regressions (*main model*) revealed that physician visits increased with increasing neuroticism and conscientiousness, whereas extraversion, openness to experience, and agreeableness did not affect physician visits in a significant way. The effect of self-rated health on physician visits was significantly moderated by neuroticism. Moreover, fixed effects regressions revealed that the probability of hospitalization in the past year increased with increasing extraversion, whereas the other personality factors did not affect this outcome measure significantly.

In further FE regression analysis (*additional model* where disability was replaced by the number of chronic conditions), physician visits increased with increasing neuroticism, whereas the effect of conscientiousness on physician visits disappeared. Moreover, the probability of hospitalization in the preceding year increased with increasing extraversion and additionally with increasing neuroticism.

### Previous research

In our study, increasing extraversion led to a higher likelihood of using hospital care. Nettle [36] also found extraversion to increase the likelihood of hospitalization for British adults aged 18–78 years. Equally, for an older sample of persons aged ≥65 years, Chapman and colleagues [37] confirmed this finding for the more specific outcome of emergency department use. In contrast to these findings, Friedman and colleagues [38] did not find extraversion to be associated either with emergency department or hospital use in general. For primary care services, Friedman and colleagues did not find extraversion to be associated with any type of service use. Pandhi and colleagues [39] showed that extraversion was related to the use of preventive health care services. Namely, higher extraversion was associated with lower use of cholesterol testing.

There are several possible explanations for increased hospital care use resulting from increasing extraversion, e.g., persons with high levels of extraversion might benefit from the ability of acting decisively in the face of urgent health problems [37] and higher extraversion has been found to increase individuals’ expectancies of positive health behavior outcomes [40]. Thus, the optimistic view on possible effects of using health care services might have increased hospital care use in our study by more extraverted persons. However, it is difficult to explain why the associations only occurred for hospital care and not for outpatient physician visits.

Based on the assumption that severe health problems are usually treated in hospitals, we have some highly speculative possible explanations, which we cannot test empirically. Maybe, (i) the ability to act decisively in the face of urgent health problems and (ii) an optimistic view of health effects of treatment in extraverts is much more pronounced when considering more severe health problems. These more severe health problems are, as already mentioned above, typically treated in hospitals. Another explanation might be that increases in extraversion are particularly associated with (iii) increases in serious accidents (e.g., traffic accidents [41]) in our dataset, which might result in hospitalization. Further research, for example based on a qualitative approach, might continue to investigate the role of extraversion and health care use.

While we found that increases in neuroticism were associated with increases in physician visits, changes in neuroticism were not related to hospitalization in the main model. However, it was significantly associated with hospitalization in the additional model. Our findings are difficult to compare with previous studies since most of the existing studies found a relationship between neuroticism and an increased *mental* health service use [6, 42–45]. In other areas of health care services, van Hemert and colleagues found an association between neuroticism at baseline and medication use as well as physician treatment nine years later in a sample of middle-aged women [46]. Furthermore, Friedman and colleagues found an association between neuroticism and the use of emergency departments, whereas they found no association between neuroticism and any hospital in community-dwelling individuals aged 65 and above.

An increase in neuroticism might be associated with participating in poor health behaviors [38]. Furthermore, higher neuroticism is associated with increased negative emotions [47]. Negative emotions such as anxiety and depression are in turn related to numerous adverse health outcomes [48]. Additionally, higher neuroticism is associated with ineffective management of stress [49], which in turn is linked to adverse physical and mental health outcomes [50]. These factors (poorer health behavior, more negative emotions and worse coping with stress) might explain our findings, i.e., the fact that physician visits increased with increases in neuroticism. However, the fact that increases in neuroticism were *not* associated with changes in the probability of hospitalization might be explained by the fact that increases in neuroticism do not reflect increases in illnesses that require hospitalization. Consequently, we assume that the increase in physician visits caused by increases in neuroticisms does not affect the rate of referral to hospital by general practitioners/specialists. Nevertheless, further research is required to explain why physician visits increase with increasing neuroticism, but increases in hospitalizations were associated with increased extraversion.

Initially, we hypothesized that conscientiousness is negatively associated with health care use because of the association between conscientiousness and accidents (negative) as well as health-related behavior (positive). Moreover, a recent cross-sectional study [7] has shown that conscientiousness is negatively related to any custodial nursing home use and emergency department visits. However, while our main regression model (2005, 2009 and 2013) showed that physician visits increased with decreasing conscientiousness, this association disappeared in additional models (2009 and 2013) where we used two waves. This might be mainly explained by the differences in time horizon, statistical power, and the differences in quantifying morbidity. The longitudinal association between conscientiousness and physician visits (main model) might be mainly explained by the aforementioned health-related behaviors associated with conscientiousness (e.g., alcohol use, smoking behavior, physical activities, or drug use [22]).

The other two personality factors (openness to experience, and agreeableness) were analyzed in an explorative manner. Indeed, none of these two factors has a robust significant effect on our outcome measures. As for our control variables (predisposing factors, enabling resources, need factors), our results are mainly in accordance with previous studies [51, 52].

In total, our results are difficult to compare with previous studies. These studies were mainly based on cross-sectional data or used a static set of personality factors to predict subsequent health care use. Contrarily, we assumed that personality varies over time. Consequently, personality was included as a time-varying variable in our statistical models. This is important, as studies have shown that personality changes over time [13, 53–55]. By using the intraindividual variation over time, our study can extend the knowledge about changes in personality and their effect on health care use.

### Strengths and limitations

Our study provides insights into the impact of personality on health care use, extending previous knowledge about associations between personality and health care use. This is the first study investigating the long-term impact of personality on health care use longitudinally. Data were derived from a population-based longitudinal study of German household (GSOEP) comprising a large sample. A panel data method (FE regression analysis) was used so that we were able to control for time-constant (observed and unobserved) factors, leading to consistent estimates (under the strict exogeneity assumption). Furthermore, the validity of self-reported admission data for hospital admission (12 months periods) was recently confirmed by Seidl and colleagues [56]. Following recommendations [57], a 3 months recall period for physician visits was used. Thus, while the recall bias is negligible, this recall period might be appropriate to capture many events.

For reasons of data availability, lifestyle factors such as alcohol consumption or smoking behavior were excluded. This might explain why Pseudo-R^2^ values were rather low. Furthermore, it is worth noting that Pseudo-R^2^ values without personality factors were only slightly smaller than Pseudo-R^2^ values with personality factors (.046 vs. .048). Also for reasons of data availability, we only used the total number of physician visits. However, it is most likely that the predictors vary by medical specialty. Moreover, even though the BFI-S showed acceptable psychometric properties, it has shortcomings for agreeableness [29]. The possibility of reverse causation cannot be ruled out in the current study. For example, it might be the case that greater illness (indicated by more physician visits) affect neuroticism. However, statistical models dealing with endogeneity (panel instrumental variable approaches) rely on strong assumptions. When instruments are weak, they lead to highly biased estimates. Therefore, an FE approach was used.

### Conclusion and future research

Our findings suggest that changes in neuroticism are associated with changes in physician visits and that changes in extraversion are associated with the probability of hospitalization. Thus, developing interventional strategies should take into account personality factors. Therefore, efforts to intervene in changing neuroticism might have beneficial effects for the health care system [58]. In total, our findings might supplement the developments in personalized medicine. Personalized medicine stratifies patient groups, for example, based on demographic or biomedical characteristics, in order to treat these groups individually and separately. Our study implicates that personality and changes in personality traits might be important target factors personalized medicine might use to discriminate different patient groups as they are directly linked to health care behavior.

Our findings furthermore indicate that ‘personality’ is an important variable for determining health care use. Thus, the Andersen Behavioral Model should incorporate ‘personality’, which would probably improve the explained variance in empiric studies.

Future research might provide evidence not only on health care use but also on the associated health care costs related to personality. Both a broader definition of health care use, distinguishing between mental health-related use and others, and a validation by using other measurements of personality (such as NEO Personality Inventory revised, NEO-PI-R) [10, 59] might provide further insights into the role of personality on health care use.

## Supporting information

S1 Table Predictors of physician visits. Results of fixed effects poisson regression (Wave 2005, wave 2009, and wave 2013).
(RTF)

S2 Table Predictors of hospital stay. Results of conditional fixed effects logistic regression (Wave 2005, wave 2009, and wave 2013).
(DOCX)
