# Supplementary material for: The role of personality in health care use: Results of a population-based longitudinal study in Germany
Source: PLoS One. 2017 Jul 26;12(7):e0181716. doi: 10.1371/journal.pone.0181716 (PMC5528826; doi:10.1371/journal.pone.0181716)
Supplement: S1 Table — Results of fixed effects poisson regression (Wave 2005, wave 2009, and wave 2013). (RTF) [file pone.0181716.s001.rtf]

S1 Table. Predictors of physician visits. Results of fixed effects poisson regression (Wave 2005, wave 2009, and wave 2013). 

	Independent variables	Physician visits	
			
Predisposing factors	Age (in years)	-0.00737***	
		(0.00198)	
	Other marital statuses (Ref.: Married, living together with spouse)	0.0489	
		(0.0343)	
	Medium education (ISCED-97, Ref.: Low education)	-0.0463	
		(0.0590)	
	High education (ISCED-97, Ref.: Low education) 	0.0141	
		(0.0852)	
	Employment status (Ref.: Currently unemployed)	-0.0380	
		(0.0411)	
Enabling resources	(Log) equivalence income	-0.0104	
		(0.0291)	
Need factors	Self-rated health (from 'very good' to 'bad')	0.423***	
		(0.0117)	
	Severely disabled (Ref.: Not severely disabled)	0.163***	
		(0.0359)	
Personality	Neuroticism (based on z-scores; higher values indicate higher neuroticism)	0.0501***	
		(0.0115)	
	Extraversion (based on z-scores; higher values indicate higher extraversion)	0.00423	
		(0.0121)	
	Openness to experience (based on z-scores; higher values indicate higher openness)	-0.0162	
		(0.0114)	
	Agreeableness (based on z-scores; higher values indicate higher agreeableness)	0.0121	
		(0.0108)	
	Conscientiousness (based on z-scores; higher values indicate higher conscientiousness)	-0.0261*	
		(0.0114)	
			
	Observations	37,185	
	Number of Individuals	14,462	
Comments: Poisson regression coefficients were reported; Cluster-robust standard errors in parentheses; *** p<0.001, ** p<0.01, * p<0.05, + p<0.10
