# Supplementary material for: The role of personality in health care use: Results of a population-based longitudinal study in Germany
Source: PLoS One. 2017 Jul 26;12(7):e0181716. doi: 10.1371/journal.pone.0181716 (PMC5528826; doi:10.1371/journal.pone.0181716)
Supplement: S2 Table — Results of conditional fixed effects logistic regression (Wave 2005, wave 2009, and wave 2013). (DOCX) [file pone.0181716.s002.docx]

S2 Table. Predictors of hospital stay. Results of conditional fixed effects logistic regression (Wave 2005, wave 2009, and wave 2013).

|  | Variables | Hospital |
| --- | --- | --- |
|  |  |  |
| Predisposing factors | Age (in years) | 1.024*** |
|  |  | (1.012 - 1.036) |
|  | Other marital statuses (Ref.: Married, living together with spouse | 1.170 |
|  |  | (0.969 - 1.413) |
|  | Medium education (ISCED-97, Ref.: Low education) | 0.796 |
|  |  | (0.509 - 1.244) |
|  | High education (ISCED-97, Ref.: Low education) | 1.320 |
|  |  | (0.692 - 2.515) |
|  | Employment status (Ref.: Currently unemployed) | 0.993 |
|  |  | (0.791 - 1.247) |
| Enabling resources | (Log) equivalence income | 0.852* |
|  |  | (0.727 - 0.999) |
| Need factors | Self-rated health (from ‘very good’ to ‘bad’) | 1.670*** |
|  |  | (1.564 - 1.784) |
|  | Severely disabled (Ref.: Not severely disabled) | 1.249* |
|  |  | (1.047 - 1.491) |
| Personality | Neuroticism (based on z-scores; higher values indicate higher neuroticism) | 1.053 |
|  |  | (0.988 - 1.122) |
|  | Extraversion (based on z-scores; higher values indicate higher extraversion) | 1.085* |
|  |  | (1.014 - 1.161) |
|  | Openness to experience (based on z-scores; higher values indicate higher openness) | 0.935+ |
|  |  | (0.874 - 1.000) |
|  | Agreeableness (based on z-scores; higher values indicate higher agreeableness) | 1.029 |
|  |  | (0.969 - 1.093) |
|  | Conscientiousness (based on z-scores; higher values indicate higher conscientiousness) | 0.965 |
|  |  | (0.908 - 1.026) |
|  |  |  |
|  | Observations | 10,424 |
|  | Number of Individuals | 3,947 |
|  | Pseudo R² | 0.048 |

Comments: Odd Ratios were reported; 95% Confidence intervals in parentheses; *** p<0.001, ** p<0.01, * p<0.05, + p<0.10
